# Participatory process to design community-driven solutions for reducing antibiotic use in chicken production in Vietnam

**DOI:** 10.1371/journal.pone.0335184

**Published:** 2025-10-28

**Authors:** Chloé Bâtie, Luong Hung Nam, Anh Ta Phuong, Trang Thi Pham, Sophie Molia, Phuc Pham Duc, Flavie Goutard

**Affiliations:** 1 ASTRE, CIRAD, INRAE, University of Montpellier, Montpellier, France,; 2 Institute of Environmental Health and Sustainable Development, Hanoi, Vietnam; 3 ASTRE, CIRAD, Hanoi, Vietnam; 4 Vietnam National University of Agriculture, Hanoi, Vietnam; 5 International Livestock Research Institute, Hanoi, Vietnam; 6 Department of Animal Husbandry, Faculty of Veterinary Science, Chulalongkorn University, Bangkok, Thailand; 7 P-lab Vietnam Animal Healthcare, Hanoi, Vietnam; 8 Faculty of Animal Science and Veterinary Medicine, Thai Nguyen University of Agriculture and Forestry, Thai Nguyen, Vietnam; 9 Center for Public Health and Ecosystem Research (CENPHER), Hanoi University of Public Health, Hanoi, Vietnam; 10 National Institute of Animal Science, Hanoi, Vietnam; 11 National Institute of Veterinary Research, Hanoi, Vietnam; University of Zambia School of Veterinary Medicine, ZAMBIA

## Abstract

International organizations emphasize the urgent need to reduce antibiotic use to combat antimicrobial resistance, including in livestock farming. Technical, regulatory, and awareness-raising strategies exist, but they often fail due to a misalignment with farmers’ realities. We hypothesize that actively engaging communities in the design of solutions will more effectively reduce antibiotic usage. We have therefore adapted and applied the ImpresS *ex ante* approach (impact of research in the South), to co-design solutions with stakeholders from the chicken and veterinary value chain at a local level in Vietnam. Eighteen participants (chicken farmers, drug sellers’ representatives, public and private veterinarians, a chicken retailer, and academic staff), working at the communal, district, or provincial level, were involved in three half-day workshops organized in Thai Nguyen province in April 2022. Through this participatory process, participants collectively envisioned a 10-year future with reduced antibiotic use in chicken farms. They identified barriers including the lack of outlets for organic meat products, lack of knowledge and awareness of biosecurity and organic farming, low compliance of small-scale farms with biosecurity, and lack of science and technology related to alternative products. Participants decided to address “knowledge gaps” barrier. They have designed two strategies to improve the training of farmers and drug sellers, so that it is closer to the chicken value chain realities and reaches a greater audience. In this study, we identify systemic barriers to reducing antibiotic use, while recommending practical solutions. We also advocate the need to include locally-developed solutions in the national action plan on antimicrobial resistance in Vietnam and to involve policy-makers in participatory processes to design effective strategies.

## Introduction

Antimicrobial resistance (AMR) is a worldwide threat responsible for both human and animal deaths [1–3] that could lead to increased economic vulnerability for farmers if nothing is done in the coming years [4,5]. One of the main drivers of AMR is the overuse or misuse of antimicrobials in human and animal health [6], which is driven by a range of biological but also cultural, economic, regulatory, technical, and sociological factors [7]. Thus the international community recognized the need to tackle this issue through global collaboration between countries and sectors [8,9] and the Global Action Plan on AMR by the Quadripartite was issued in 2015 [10]. In this plan, recommendations, including strengthening global governance, financing, surveillance, prevention, and research and development, have been developed, especially in low- and middle-income countries (LMICs), where interventions are particularly needed.

Because antimicrobial usage (AMU) is context-dependent, there is no universal or one-size-fits-all solution to reduce or improve the use of antimicrobials. Studies conducted in Europe have widely explored technical solutions, including probiotics, vitamins, vaccines, or better biosecurity, as ways to reduce AMU [11]. Other studies recommend raising the awareness of farmers and drug sellers on AMU, AMR, or alternatives [12], as lack of knowledge of farmers is often pointed out as a major driver of AMR [13,14]. In LMICs, policy development is often advocated to better regulate antimicrobial access, use, and quality [15–17]. Other common recommendations concern better veterinary services and access to antimicrobials [18], food safety issue management [19], increased biosecurity, and better training access for farmers [15]. However, such measures, including regulations, depend on the country in which they are implemented and must be adapted to the context to be effective [20]. As argued by Ducrot et al., policies must be adapted to the political and social context and thus developed by including actors in finding solutions to their problems [15].

To address this complexity, many researchers now recommend studying AMR not only from an individual perspective but by using systemic approaches to consider the socio-ecosystem [21–24]. These approaches are transdisciplinary and multi-sectorial to ensure better health for humans, animals, and the environment in addressing complex one health issues [25]. Interactions between sciences and society are taken into account, and stakeholders can be included from the identification of problems to the implementation of solutions [26]. To that effect, participatory approaches allow stakeholders to design their solutions, adapted to their context, to reduce the usage of antibiotics [27,28]. An added value to these processes lies in the fact that they allow innovative approaches to emerge by bringing new perspectives on an issue [29].

In Vietnam, chicken production is an evolving and growing sector [30]. Consumers’ habits have changed in the last decade, and there is now an increasing demand for animal protein, particularly chicken meat [31]. The sector, traditionally represented by household or small family commercial farmers selling to live-bird markets [32,33], is progressively shifting toward intensive practices under the push of the government [34]. A rising number of consumers, especially in urban centres, feel concerned about food safety issues [31], and buying meat in supermarkets has become more common. However, AMR represents an important issue in this sector that could threaten its development. Bacteria resistant to critical antibiotics have been observed in farms [35] and farmers report needing to increase antibiotic dosages to maintain efficacy [36]. Vietnamese chicken farmers are generally heavy users of antibiotics [37,38]. Double dosage, purchase without prescription, no or little veterinary advice, preventive use, and no respect for the withdrawal time are all common practices of some family commercial farmers in Vietnam to prevent and treat disease, which in turn, contribute to the emergence of resistant bacteria [39–42].

To fight against AMR, Vietnam has adopted two successive National Action Plan (NAP) in 2017 and 2021 in the livestock sector [43,44] that was followed by the promulgation of new regulations to reduce AMU. Other solutions to mitigate the AMR risk have been developed such as raising awareness through the organization of workshops by private or public stakeholders or developing model farms in biosecurity. However, a qualitative study conducted in 2023 to analyse the perceived impact of the NAP implementation in Vietnam by the public at the community level found that even though major advances have been made, like regulatory strengthening, it was necessary to better involve communities in order to raise awareness about AMU so that these regulations would be effective. Indeed, the study explains that in Vietnam the decision to use antibiotics is driven by social or economic responses [45].

We thus hypothesized that effective solutions must be codeveloped by local stakeholders themselves. This study aimed to involve relevant stakeholders, from the chicken and veterinary drug value chains, to design strategies to reduce the usage of antibiotics in chicken production in Vietnam using a participatory approach, the ImpresS *ex ante* approach [46] through a series of three workshops. The objective of this methodology is to build a collective reflection on the changes that a group of stakeholders wish to observe to respond to their problem based on their active participation.

## Materials and methods

### Context of the workshops

The workshops took place within the H2020 European project ROADMAP (Rethinking of Antimicrobial Decision-systems in the Management of Animal Production) that aimed to foster transitions towards prudent use of antimicrobials in different production systems to manage AMR (https://www.roadmap-h2020.eu/). The project was implemented in various countries in Europe, Mozambique, and Vietnam. In Vietnam, a first study was conducted in the north (Hanoi province) and south (Lang An province) to develop a typology of chicken farms [47]. Three main production systems were identified: intensive, family commercial, and household farms with the first two using antibiotics both in prevention and treatment whereas the last one used antibiotic only to treat their chickens. A second study contributed to map the veterinary drug value chain through stakeholders’ interviews in the south and north of the country [48]. Finally, a last survey conducted in north Vietnam (Thai Nguyen Province) with farmers and veterinarians explored, through a systemic approach, the transition process of chicken farmers toward a lower usage of antibiotics [36]. Locally developed solutions were identified such as using local hand-made probiotics to produce “quality” chickens with fewer antibiotics, as well as disseminating advice from “champions” of antibiotic use (ABU) reduction. We also highlighted local networks (cooperatives, relationships with veterinary services, and influential veterinarians) as drivers to reduce ABU.

Based on this last study, we involved some of the participants already undertaking a change of practices and recruited new ones, in a participatory process to engage these stakeholders in the design of community-driven solutions to reduce ABU in chicken production. In this work, the term strategy refers to a set of measures that enable solutions to be implemented by identifying the actions to be taken and the actors who will take these actions. A solution refers to a response to a given problem, without prejudging the effectiveness of the said response.

### ImpresS *ex ante* approach

**Fig 1:**
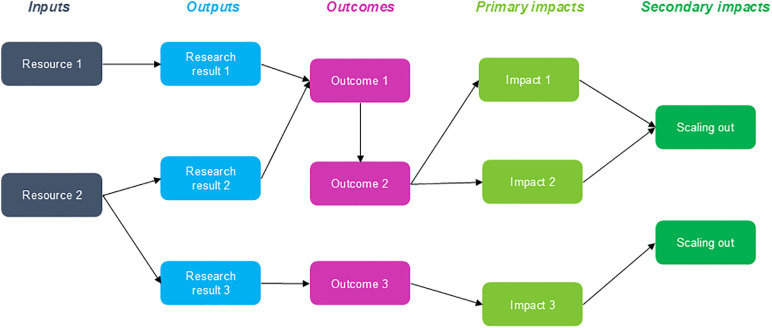
Impact pathway visualization from Blundo Canto and al., 2020 [46].

To engage stakeholders in the co-building process, we have adapted the ImpresS *ex ante* (Impact in research in the South) approach developed by Cirad [46]. This approach is based on the theory of change that highlights causal loops to explain the process of change [49]. The aim is to build an intervention through the construction of a shared vision and an impact pathway [46]. The impact pathway is the visual representation of the different steps of the implementation of an intervention and the causal links between them (Fig 1). The implementation of an intervention will contribute to long-term (second level) and short term (first level) impacts that are not the direct consequences but rather the long- and medium-term effects of it. The outcomes are the desirable changes in the actors that the intervention will contribute to, thanks to the adoption of the outputs of the intervention (products of the intervention) that are produced from inputs (resources necessary to carry on the intervention). By participating in this process, involved stakeholders, who can be decision-makers, researchers, and members of the community, can better own the outputs of the intervention. This ownership is facilitated by the understanding of the underlying mechanisms that contribute to the desired change in “practices, behaviors, and interactions” [46]. The approach is based on four main stages: mapping of desirable changes and construction of the intervention’s strategy, consolidation of the impact pathway and its translation into different products and construction of the intervention’s narrative. The process is iterative, with each step contributing to the next one, but it is also adaptable and flexible, as the approach can be tailored to the study objectives during its implementation.

### Organization of the three workshops

Participants were recruited for three workshops that were held between the 12/04/2022 and 25/04/2022 in a town of Thai Nguyen province, north Vietnam, in the same district as the prior study mentioned earlier on chicken farmers’ transition to lower ABU [36]. This commune is central, holds the district veterinary services, and was also one of the main towns of interest in our previous study [36]. The workshops were conducted in Vietnamese. They were facilitated by a researcher from Hanoi University of Public Health (HUPH) familiar with the ImpresS *ex ante* approach [27], a veterinary research assistant from the Vietnamese National University of Agriculture (VNUA) who was trained in participatory methods and facilitation by the first author, a researcher from Thai Nguyen University of Agriculture and Forestry (TUAF) in charge of the workshops’ organization and the first author. The first author received training in participatory approaches, including participatory modelling. Two observers from TUAF and VNUA were responsible for taking notes and pictures of the results (vision of the future, problem tree, outcome mapping and action plans), one student oversaw the technical setup of the workshops, and another student provided simultaneous translation Vietnamese – English to the first author. The three workshops were organized weekly to give time for a short analysis and to maintain the participants’ engagement. The workshops were recorded with a voice recorder and filmed with two cameras placed on both sides of the room.

Participants were recruited by a TUAF researcher and the veterinary district services, according to a list drawn up by the research team based on the previous study [36]. The aim was to obtain a shared representation of the issue while recognizing the multiplicity of views [50]. We therefore included public sector stakeholders operating at different levels (province, district, commune), a university professor, and private sector stakeholders. Among the latter, we included drug sellers of different categories (drug companies, drug agencies with different sizes identified as agency level 1 and agency level 2), chicken farmers of different production systems (intensive and family commercial farms – household farms were not included because they were identified as not using a lot of antibiotics in our previous study), and a chicken trader. Categories of stakeholders are described elsewhere [48]. To compare points of view to identify solutions, we also included stakeholders (drug sellers and farmers) already engaged in the change of practices identified in the previous study [36]. They represented half of the participants. When a participant couldn’t attend all three workshops, he or she was replaced by another person from the same category.

The objectives of the study were presented to the participants, and written consent was obtained before each workshop. We carefully explained the limits of the process to not create false expectations among the participants. This study was approved by the Ethics Review Board for biomedical research of Hanoi University of Public Health with the application number 021–391/ DD-YTCC. Authorization to conduct this work was given by the Sub-department of Animal Health and Livestock Production (Sub-DAHLP) of Thai Nguyen province.

### Course of the workshops

The course of the workshops was adapted from the ImpresS *ex ante* approach [27,28,46] and followed four steps: defining the common vision, building the problem tree, mapping the outcome pathway, and transforming strategies into action plans.

#### Step 1: Defining the common vision.

The first step defines the common vision, which is the ideal situation that the participants would like to achieve in 10 years (or more) with the objective to reduce and better use antibiotics in chicken production in Vietnam.

For this purpose, a first-round table discussion with all participants was conducted (icebreaker). This was followed by a discussion on the current situation regarding the use of antibiotics and antibiotic resistance in Vietnam and Thai Nguyen province. This helped to define the boundaries of the issue under study and to give the definitions of antibiotic and antibiotic resistance to ensure the same understanding for everyone.

Next, participants were asked to write on a yellow sheet of paper the vision to be achieved in ten years to reduce the use of antibiotics in chicken production in the province by answering the question: “What situation would you like to reach in 10 years with the objective to reduce and better use antibiotics?”. The different visions written by the participants were grouped into themes by the facilitators in agreement with the participants (i.e., a theme could have different visions). Each person was then invited to come and vote for the theme that they wished to be addressed in the following steps. After discussion, the vision of the future was reformulated by the research team based on the selected themes and was validated by the group.

#### Step 2: Building the problem tree.

The second step consisted of identifying problems that prevent the realization of the common vision. For this, we used the “problem tree” tool as adapted by the ImpresS *ex ante* approach [46]. The tree represents the underlying problems and the causal link between them in relation to the central issue, i.e., reducing ABU.

For this purpose, two red sheets of paper were distributed to the participants with the instruction to write only one problem per sheet. Each participant presented his or her problem(s) and posted them on the board. The problems were then grouped by theme by the research team in agreement with the participants. Then, for each theme, the problem tree was constructed progressively by successively asking the question “*Why*?” to determine the root causes of the central issue.

#### Step 3: Mapping the outcome pathway and identifying strategies.

The root causes of the central issue previously identified were then prioritized by the participants. The root with the highest number of votes was chosen to initiate the mapping of desirable changes (outcomes).

**Fig 2:**
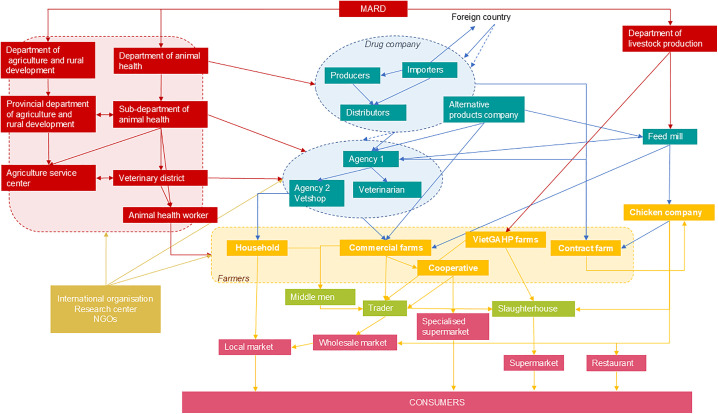
Stakeholders’ map of the chicken value chain presented to the participants during workshop 2 in Thai Nguyen province, April 2022 adapted from Bâtie et al., 2022, 2025 [47,48]. Agencies 1 and 2: local drugstores; MARD: Ministry of Agriculture and Rural Development; NGOs: Non-Governmental Organizations; VietGAHP: Vietnamese Good Animal Husbandry Practices. Red boxes: public sector; blue boxes: inputs; orange: production system; green boxes: collector and processing; purple: distribution and consumption; brown boxes: international partners.

The next step in the approach is to identify the major, influential, and impacted stakeholders of the intervention and to define their roles. As this step had already been done during previous studies during the mapping of the veterinary value chain and chicken value chain [47,48], a version of the chicken production chain in Vietnam linked to the veterinary drug distribution chain was projected in Vietnamese for validation and discussion with the participants (Fig 2).

Then, for the selected problem, the outcome pathway was mapped. This step aimed to identify the changes to be made, the actors involved in it, as well as the obstacles to these changes. Finally, strategies to overcome these obstacles were formulated. To identify the changes, we asked the question “*Who needs to change and how to address this problem?*”. The changes identified were written on an orange sheet by the research team while the stakeholders involved were written on a green sheet. The new obstacles corresponding to the changes were written on a red sheet of paper and the strategies for responding to these obstacles on a blue sheet.

#### Step 4: Transforming strategies into action plans.

After validation of the identified strategies, the participants were separated into two balanced groups previously constituted by the research team (so that each category of actors was represented). Each group was assigned a strategy and was asked to build an action plan to implement it by answering the following questions (posted in the room in Vietnamese): *Who* (define the target of the intervention); *What* (define the content of the intervention); *By whom* (define the actors carrying out the intervention); *When* (define the timing of the intervention); *Where* (define the location of the intervention); *How* (define the format of the intervention). Each group presented its action plan during a plenary session for discussions and feedback.

### Data analysis

The three workshops were transcribed in Vietnamese and entirely translated into English on Microsoft Word by a professional in library science. Results of the workshops such as the vision of the future, problem tree, outcome mapping, and action plans were documented through photographs and reproduced using PowerPoint software. Themes that emerged from these results were identified in the transcripts. This step helped us to correct and tweak the visions of the future, problem tree, outcome mapping, and action plans, to explain the opinion of the participants regarding these themes and the connection between participants’ ideas. After each workshop, results were clarified, reformulated, analyzed, and discussed by the research team to produce a summary of the previous activities for the participants at the beginning of each workshop. Participants validated the results and were free to modify and complement them. The impact pathway mapping was done by the research team at the end of the three workshops (short-term and long-term impacts came from the analysis of the transcripts and the common vision) and was presented to the participants for discussion during the restitution meeting organized in February 2023.

## Results

### Organization of the workshops

In total, eighteen people participated in the workshops, with fifteen attending each session. Three participants attended only the first two sessions and were replaced by others from the same category for the final workshop. Five participants were from the public sector: two from the provincial veterinary services (Sub-DAHLP), one from the district veterinary service, one communal veterinarian, and one professor from TUAF. Ten people represented the private sector, including six farmers: one integrated farmer, and five family commercial farmers, two of whom were from the same cooperative producing chickens with lower ABU as described in the study on the transition process of farmers toward lower ABU [36]. Among the three drug sellers, one represented a pharmaceutical company that specialized in alternative products, while the others were from a first and second-level agency (which differ by their source of supply and their target customers). Finally, one of the participants was a local chicken retailer. The sociodemographic characteristics of the participants and their participation to the previous study are summarized in Table 1. The workshops consisted of 8 men and 7 women aged between 18 and 25 (n = 1), 26 and 40 (n = 7) and 40 and 65 years old (n = 7).

**Table 1:** Sociodemographic characteristics of the three workshop’s participants, April 2022, Thai Nguyen province, Vietnam.

Participants	Sector	Profession	Category	Gender	Participation previous study^†^	Attendance		
						W1	W2	W3
Participant 1	Public	Veterinary services	Provincial	Male	No	X	X	
Participant 1bis	Public	Veterinary services	Provincial	Male	No			X
Participant 2	Public	Veterinary services	Provincial	Female	No	X	X	
Participant 2bis	Public	Veterinary services	Provincial	Female	No			X
Participant 3	Public	Veterinary services	District	Female	No	X	X	X
Participant 4	Public	Veterinary services	Communal	Female	Yes	X	X	X
Participant 5	Public	Academia	Professor	Male	No	X	X	X
Participant 6	Private	Farmer	Integrated	Male	Yes	X	X	
Participant 6bis	Private	Farmer	Integrated	Male	Yes			X
Participant 7	Private	Farmer	Family commercial*	Male	Yes	X	X	X
Participant 8	Private	Farmer	Family commercial*	Male	Yes	X	X	X
Participant 9	Private	Farmer	Family commercial	Female	No	X	X	X
Participant 10	Private	Farmer	Family commercial	Female	No	X	X	X
Participant 11	Private	Farmer	Family commercial	Male	Yes	X	X	X
Participant 12	Private	Trader	Trader	Female	No	X	X	X
Participant 13	Private	Drug seller	Pharmaceutical company	Male	No	X	X	X
Participant 14	Private	Drug seller	Agency 1	Male	Yes	X	X	X
Participant 15	Private	Drug seller	Agency 2	Female	No	X	X	X

W: workshop; bis: participant replacing an absent participant from the same category.

^†^: Participation to the study on the transition process of chicken farmers toward lower antibiotic use [36].

*: Members of the same chicken cooperative producing chickens with lower antibiotic use as described in the study on the transition process of chicken farmers toward lower antibiotic use [36].

The workshops lasted an average of 3 hours, including a break for informal discussions. Two facilitators conducted the sessions: one led the discussion, while the other wrote on the board. During the second workshop, one of the facilitators was replaced by another member of the team.

### Course of the workshops

#### Step 1: Defining the common vision.

Participants identified five main objectives that they would like to achieve in the next 10 years to reduce and improve the use of antibiotics locally: 1) improve access to alternative treatments such as herbs, probiotics, or natural products; 2) raise farmers’ awareness on science and technology and the adverse effects of antibiotic resistance; 3) transition from small-scale to large-scale chicken farming; 4) control and manage antibiotic production and market distribution; 5) improve biosecurity on farms and develop organic farming. Points 1, 2, and 5 were included in the joint vision that was validated by the participants which reads as follows:

*“In 10 years, we would like to see farms with good biosecurity conditions and organic farms using alternative products, as well as training for farmers on animal husbandry techniques and the drawbacks of antibiotic resistance”*

#### Step 2: Building the problem tree.

**Fig 3:**
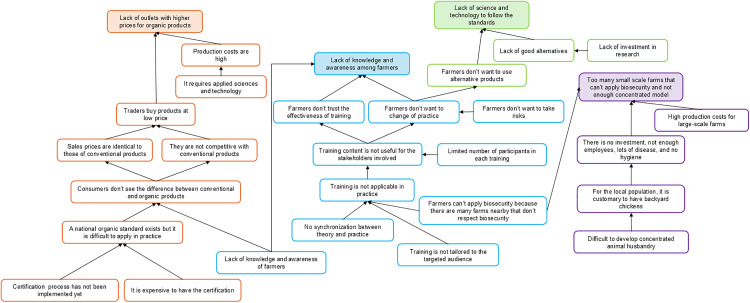
Problem tree built during the first workshop including 30 identified problems, Thai Nguyen province, April 2022. The four colours, orange, blue, green, and purple, correspond to the four roots of the problem tree respectively: “Lack of outlets with higher prices for organic meat products”, “Lack of knowledge and awareness among farmers”, “Lack of science and technology to follow the standards”, and “Too many small scale farms that can’t apply biosecurity and not enough concentrated model” (“concentrated model” refers to intensive farms that apply biosecurity, produce in batches and control the inputs and outputs).

Participants identified four mains branches that prevent the achievement of the common vision with four roots that were: lack of outlets for organic meat products, lack of knowledge and awareness of biosecurity and organic farming, low compliance of small-scale farms with biosecurity, and lack of science and technology related to alternative products such as probiotics and yeasts. These branches included a total of 30 problems (see Fig 3).

**Lack of outlets for organic meat products:** The production of organic chickens was identified as expensive and complicated to set up for farmers.

First, a participant explained that a national organic standard for organic meat products (n°11041–12/2017) existed but was still not enforced because not adapted to the Vietnamese context. Even if the creation of a national standard is being discussed at the national level, the participant added that the certification process would be too costly for farmers, and there would be a shortage of certification bodies in Thai Nguyen province.

The other reason cited was the lack of organic feed in Vietnam complying with international standards to be able to produce organic chickens. Participants explained that for the time being, farmers could only aim for this type of production (i.e., reducing their usage of antibiotics, use probiotics) but not achieve it. Another problem reported by the participants was the lack of profit for the farmers when producing “organic” chickens (not meeting international standards but with no or little usage of antimicrobials and usage of natural products). The production costs were identified as higher, while the chickens were still bought by retailers for the same price as conventional chickens., The main reason of it being that consumers were not yet ready to spend more money on better quality products. Indeed, according to participants, consumers see no visible difference between “organic” chickens and conventional chickens on the local market due to the lack of packaging and certification. They are therefore unwilling to pay a higher price for them.

“We have not proven it [organic farming], and we have not made differences between what is organic and what is non-organic? It is our awareness; we still have not done as a standard. We have not had a set of standards, and have not announced the quality of our products, so the real and fake products [organic and conventional] are competing together. Therefore, the people who produce the real products feel too hard and the people who make fake things still exist. That is the reason why it is difficult.” (Workshop 1, chicken farmer)

**Lack of training and awareness of biosecurity and organic farming:** This problem’s root was linked to two main issues: farmers do not want to change by fear of risk, and lack of confidence in training programs.

Participants noted that many farmers resist change, because they believed in their experience and their habits. The pharmaceutical company representative stressed that during seminars, farmers were more interested in disease diagnosis learning than improving their farming practices.

“People don’t want to change, don’t want to discover new things and only follow their old ways.” (Workshop 1, chicken farmer)

Our respondents highlighted that training participants usually did not trust the training courses because their content was considered not adapted to the field realities. Recommendations were therefore inapplicable in practice and not useful according to them. Respondents working in veterinary services also recognized that these programs had limitations, such as not being able to reach everyone. The alternative products company representative also mentioned that pharmaceutical companies were focusing too much on explanations of how to treat rather than preventing diseases and thus were giving insufficient advice on biosecurity to farmers. A chicken farmer explained his perceived main constraints of such training in three points:

“Firstly, that is the mismatch between theory and practice. After taking part in the training course, people don’t follow the guide on your farm because their facility conditions are too weak, they have not been properly invested to be able to apply the techniques they get from the training sessions, for example. The second reason is that they wonder if they do that while their neighbors don’t, so diseases still happen or the production cost increases while the price of chickens does not increase compared to others, so they also lose their minds. The third is about the high density of chickens.” (Workshop 1, chicken farmer)

Drug sellers were also said to be insufficiently knowledgeable and lacked the will to enable a change in practices. Indeed, a large part of their income was still dependent on antibiotic sales and was facilitated by the absence of mandatory prescriptions or diagnostic tests before selling antibiotics.

“Veterinarians do not have many practical problems after graduating from school. When farmers ask for their medicines, they only care about selling products and don’t pay attention to raising farmers’ awareness about medicines.” (Workshop 2, pharmaceutical company representative)

**Low compliance with biosecurity of small-scale farmers:** Small-scale farmers, typically raising between 50–100 chickens and 3–5 pigs, were identified as a problem to reduce ABU in Vietnam. According to the participants, their lack of knowledge prevents them from implementing good biosecurity measures. In addition, they were concentrated in one area with free-running chickens that could spread diseases. Low biosecurity was accentuated by the proximity between farms and houses with people moving from one farm to another without applying any preventive measure to reduce disease transmission. Because not all farmers were paying attention to disease transmission, the other farmers did not want to apply biosecurity alone. Investments were also very limited, and farmers did not hire employees who could help them implement these measures. According to the participants, keeping a small number of roaming chickens is related to the customs of the country and prevents the development of intensive farming.

“According to the traditional farming practice of the Vietnamese people, 70% of livestock production is still household farming with a small scale. They only raise 50-100 chickens and 3-5 pigs. With that small scale, they cannot apply biosecurity in farming. Biosecurity requires synchronization from the breed, feed, input, output, the process of disease prevention and the methods of managing the farms as well, while they won’t have enough employees to apply biosecurity.” (Workshop 1, unknown participant)

**Lack of science and technology related to alternative products:** This final branch refers to the situation in Vietnam where the safety and therapeutic or preventive efficacy of alternative products have not been scientifically tested; alternatives products are made in a more traditional, handcrafted way than conventional veterinary drugs manufactured in factories. Because of that, farmers cannot comply with the international organic standards.

Barriers to the adoption of new techniques (science and technology) were the poor quality of alternative products and the fear of change of farmers and drug sellers. Alternative products did not show as effective results as was the case for antibiotics. Indeed, farmers and drug sellers were identified as looking for immediate results without prior diagnosis, which is made possible by antibiotics. In addition, farmers lacked knowledge of how to use alternative products and were not sufficiently guided in their use.

#### Step 3: Mapping the outcome pathway and identifying strategies.

Participants chose to address the following root: “lack of training and awareness on biosecurity and organic farming”. Indeed, this problem was raised several times during the construction of the common vision and the problem tree. The second choice related to small-scale farms that did not respect biosecurity. There were also many discussions on organic production.

**Fig 4:**
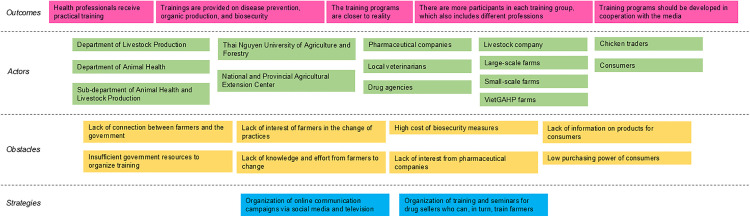
Outcome mapping visualization drawn during the second workshop to address the problem “lack of training and awareness on biosecurity and organic farming”, Thai Nguyen province, April 2022. Pink boxes: outcomes (desirable changes); green boxes: actors involved in the changes; orange boxes: obstacles to changes; blue boxes: strategies. VietGAHP: Vietnamese Good Animal Husbandry Practices.

During the second workshop, they drew the outcome mapping to solve this problem, which is represented in Fig 4.

The main changes identified were providing practical training for health professionals (students, veterinarians, and drug sellers), developing training programs that were closer to reality, providing training on disease prevention, organic production, and biosecurity, increasing the number of participants per training course with groups including different professions to promote knowledge exchange, and finally cooperating with the media to have a wider reach.

“In my opinion, I also want the training sessions to transmit livestock techniques as well as scientific and technical advances to farmers. I hope that those techniques are not only in theory, but they are as close to reality as possible so that people can apply them in practice.” (Workshop 2, chicken farmer)

“In my opinion, training sessions should have both agents [veterinarians and drug sellers] and farmers so that everyone can understand problems, but it will not be effective if those sessions have only farmers or only dealers.” (Workshop 2, drug seller)

Improving training and knowledge would allow a gradual transformation of production in the region towards organic production of local breed chickens or “hill chickens”. The objective would be to follow the Vietnamese Good Animal Husbandry Practices (VietGAHP) standard (many farmers in the region were now certified). The Sub-DAHLP and the agricultural extension centres should offer courses on organic production so that people understand in depth what it is and learn how to master it.

The stakeholders that the participants have identified as having a role in the strategy were the public sector, the Department of Livestock Production, the Department of Animal Health, Thai Nguyen University of Agriculture and Forestry (including the faculty of veterinary medicine), National and Provincial Agricultural Extension Centres, and the Sub-Department of Animal Health and Livestock Production. For health professionals (private sector), they identified drug agencies, local veterinarians, and pharmaceutical companies. For farmers, they identified VietGAHP certified farms, small-scale farms, large-scale farms, and livestock companies. Finally, the other stakeholders were chicken traders and consumers.

Obstacles to change included a lack of connection between farmers and government, lack of interest of farmers in change of practices, lack of knowledge and effort from farmers to change, insufficient government resources to organize training, and lack of interest from pharmaceutical companies which were only interested in best-selling antibiotics and disease treatments, high cost of biosecurity measures, low purchasing power of consumers, and lack of information on products for consumers. The participants also stressed that this change in consumption habits would take place gradually, as for the time being consumers were still not convinced of the quality of the chickens sold in supermarkets (they do not know where they come from, whether they are sick, etc.).

Two strategies were identified to overcome these obstacles. Firstly, the organization of online communication campaigns via social media and television targeting farmers and consumers (strategy 1). Secondly, the organization of trainings and seminars for drug sellers who can in turn train farmers (strategy 2).

“The company has another solution, which is to film videos about raising awareness of vaccines, farming procedures, or diagnosing and treating procedures. In addition, other companies have already had that type of video, it’s very easy to find them online, especially on Youtube. They filmed those videos without seminars, so people can watch them whenever they want.” (Workshop 2, government representative)

#### Step 4: Transforming the strategies into action plans.

To address the issue of training but also the lack of awareness on alternative products, organic production and biosecurity, participants designed two action plans during the last workshop.

**Action Plan 1 (strategy 1):** To reach more people, communication programs should be disseminated through social media or television via short videos. To ensure that these videos meet the expectations of the people concerned, surveys to assess the needs of farmers should be conducted beforehand. The targets of these programs will be farmers and consumers (two different programs). The communication campaign will focus on organic farming, biosecurity, and alternative products but will also highlight the benefits of using fewer antibiotics and producing safer products. Programs will also include visits to some model farms for biosecurity and organic farming. They will be broadcast on national TV channels (VTC 16, VTV 2) and local TV channels twice a week at 8 p.m. when farmers are available. The second communication channel to reach the same targeted audience should be social media (Facebook, Instagram, Zalo). Funding will come from the Vietnamese government and foreign organizations. Training contents will be produced by a TV channel and be guided by the Ministry of Agriculture and Rural Development and associated agencies (Department of Livestock Production, National Agricultural Extension Centre, Department of Agriculture and Rural Development, Department of Science and Technology, TUAF).

Below is a verbatim extracted from the discussions of the group 1.

“We should introduce the topic like that. At the beginning, we must highlight the value of the post-breeding product [chicken meat]– a non-antibiotic product – so what is its value? What is its difference in comparison to a common product which uses antibiotics? The second thing is that what do we have to do to not use antibiotics in farming? We have to apply biosecurity methods, use vaccines and have other activities to prevent diseases.” (Workshop 3, unknown participant group 1)

**Action Plan 2 (strategy 2):** The second plan aimed to provide better training on biosecurity and organic production to health professionals. The targets of this program will be drug sellers (retailers and veterinarians who sell drugs at the veterinary pharmacy or directly on the farm). The topics covered will be the principles of antibiotic use in livestock production, the disadvantages of overusing antibiotics, the process of biosecurity and organic production, and advice on using alternative products. Those who will be responsible for delivering the training will be pharmaceutical companies, technicians, agricultural extension officers from agricultural service centres, and professors from veterinary faculty (in this case, TUAF), and Sub-DAHLP.

Training will be organized twice a year, but also during epidemics (training sessions on biosecurity and safety to learn how to manage new diseases), when a new alternative product appears on the market, and when new staff members join veterinary pharmacies. It will be possible to organize this training at the commune, district, or farm level (they will be able to observe the farm environment). The training will be face-to-face or distance learning. Leaflets will also be given to participants.

### Impact pathway mapping

**Fig 5:**
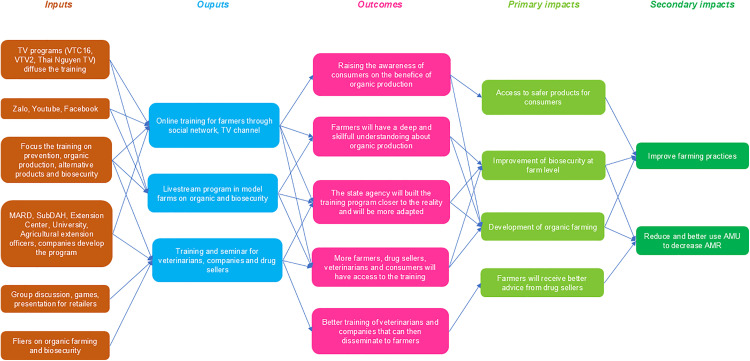
Impact pathway built by the research team from the three workshops held in Thai Nguyen province, April 2022. MARD: Ministry of Agriculture and Rural Development; SubDAH: Sub-department of Animal Health; AMU: antimicrobial use; AMR: antimicrobial resistance.

The impact pathway mapping based on the various steps of the workshops is presented in Fig 5.

## Discussion

### Co-development of strategies to reduce antibiotic use in Vietnam

In this study, we have adapted and applied the ImpresS *ex ante* approach to allow locally-based stakeholders to co-develop strategies, adapted to their context, to reduce the usage of antibiotics. Chicken farmers, drug sellers’ representatives, public and private veterinarians, a chicken retailer, and academic staff have identified the need to improve the training of farmers and drug sellers on biosecurity and organic production. Training courses could be set up through the organization of workshops in person or online using live streaming. These programs could be developed by different departments operating at the provincial level under the Ministry of Agriculture and Rural Development, in collaboration with the University of Agriculture and Forestry of Thai Nguyen, TV channels, and private sector stakeholders. The research team found the emphasis on improving training programs somewhat unexpected. Indeed, many workshops organized by international organizations and public and private sectors were already identified during our last surveys [36,48]. In a previous study [36], we identified that some farmers that were cooperatives’ members reduced ABU and that these farmers had access to probiotics (efficient at improving health outcomes, therefore likely to decrease ABU needs) through their cooperative. We thus expected that more targeted solutions, such as the model of a cooperative, would have been explored during the workshops. We could explain this by a mix of factors. First, the discussion was time-limited, participants chose quick actions rather than longer-horizon options like cooperative that could also be felt outside the group’s decision space. Second, decision biases (status-quo and present-bias) push groups toward low-effort steps and away from months of coordination needed to set up or expand a cooperative. Finally, in mixed-stakeholder workshops, participants often avoid pushing ideas that could seem self-serving. So even a cooperative representative that believes in this solution may not insist on it. Raising awareness of farmers and drug sellers is one of the objectives of the Vietnamese National Action Plan [44], the Global Action Plan, and is often described as a solution to reduce AMU in many knowledge, attitude, and practice surveys [14,51]. In a scoping review on interventions that have successfully changed AMU in livestock production, 10 interventions of the 28 identified implemented “informational and educational measures” as a strategy [52]. Improving knowledge was done either by providing education (training, work groups), which aligns with Strategy 2 designed by our participants, or through information (communication tools, sharing experience, …), which aligns with Strategy 1. The same review also showed that farmers and veterinarians were the most often targeted by the interventions as in our study. Participants therefore confirmed that raising awareness on best practices was part of the solution.

Our study also introduced innovative way to design solutions to fight antibiotic resistance (ABR) in Vietnam. Through the participatory process, we identified barriers that limit the efficiency of the existing training programs. There is a lack of trust in the effectiveness of training, as they are often considered too disconnected from the reality faced by farmers and because farmers are reluctant to change their practices. To overcome these obstacles, programs should thus be closer to field constraints, aligned with the interests of farmers, and be more inclusive. In the case of Thai Nguyen province, efforts should focus on organic farming and biosecurity. Developing organic livestock production is one of the objectives of the Vietnamese government [53]. However, to our knowledge, training in organic farming practices is not common in Vietnam. This might be due to the relatively new introduction of organic farming policies but also the absence of enforcement at the time of the study, and the lack of organic feed and possible meat outputs as explained by the participants. However, training on biosecurity exists and has demonstrated positive results in terms of ABU reduction, such as the Model Farm program developed by FAO in Vietnam [54]. Finally, participants expressed the need to reach a greater audience in the training programs by using different media (TV channels, YouTube, social media). This solution has limitations and should not be considered as a stand-alone behaviour tool as it is often not enough to change behaviour. Indeed, for a durable change, there is a need of multiple measures. For example, in the VIPARC project conducted in Vietnam in chicken production, they have achieved AMU reduction by using several tools together [55]. However, the large exposure allowed by the diffusion of videos on TV of social networks increase the likelihood of change of behaviors. A study conducted in Vietnam showed that television was the main source of information that farmers intentionally sought out for information on antibiotics and animal health, followed by direct contact with stakeholders with good practice and loudspeakers. Conferences and workshops emerged as an unintentional source of information [56]. The same study showed that better self-reported practices were associated with more time spent obtaining information. Programs broadcasted through the national TV but also on the internet are common in Vietnam and reach a diverse audience (e.g., consumers or other stakeholders) operating at the provincial or district level. Increasing the awareness of these stakeholders can add additional pressures on farmers. Finally, the multiplicity of communication channels improves the overall efficiency. Live streaming programs featuring farmers with good practices raise public awareness of ABU in Vietnam while reaching a large audience. This solution is close to stable schools implemented in the 2000s in Denmark on organic dairy farming for learning in groups based on a concrete situation [57]. Regarding online learning, another study conducted in Germany showed that veterinarians preferred multidisciplinary networks and e-learning [58].

Improving training programs at a local scale is often identified as a solution to improve ABU practice [15]. This can be done by involving provincial universities and government but also local private stakeholders. The practical training tailored to local needs must be however understood within the broader context of systemic barriers that continue to challenge the reduction of antibiotic use [59]. For instance, economic pressures and entrenched practices have historically hindered the adoption of new approaches. Our focus on localized training, which directly engages stakeholders and addresses their specific concerns, represents a critical step in overcoming these barriers, differentiating our approach from previous efforts that may have lacked this target.

Our study supports the fact that there is no one-size fits all solution to the problem of AMR. Similar workshops were conducted in France within the Roadmap project, which included representatives of national pig and poultry organizations. The vision of the future designed in these workshops expressed the need to optimize antimicrobial use (rather than solely focusing on reducing ABU). The strategy adopted consisted of designing appropriate indicators that would combine data on ABU, ABR, as well as the health and well-being of animals [28,60]. Some broad categories of problems identified were close to those in our study, such as a lack of knowledge and motivation. But, in the French case, this issue concerned mostly the lack of knowledge on the obstacles to change or the reduced motivation to sustain changes in a context where more than 50% of AMU reduction has already been achieved in the past decade. This example shows the diversity of challenges to reduce and optimize antibiotic use but also of solutions that can be designed to address AMR. Considerations on this issue depend on both the context and the participants involved in the design of solutions [61]. In Vietnam, where the regulatory framework on AMU is new compared to France, the needs and constraints are different. For example, the efforts were focused on reducing the usage of antibiotics rather than optimizing their use. This can be explained by the fact that the priority for Vietnam is first to remove antimicrobial growth promoters and prophylactic antibiotic use before selling the chicken and then focus on better antibiotic use.

### Engaging stakeholders toward a change of practice

Besides planning targeted solutions [28], the strength of participatory approaches lies on the active participation of individuals, which has multiple positive outcomes.

With this study, we showed different way to engage stakeholders and how it can contribute to a change of practice. Stakeholders engagement can be defined as “an iterative process of actively soliciting the knowledge, experience, judgment, and values of individuals selected to represent a broad range of direct interest in a particular issue, for the dual purposes of: creating a shared understanding; making relevant, transparent and effective decisions” [62]. First, participants were invited to participate in a series of three workshops. The repetitive participation in a collective thinking process increases the engagement of actors to change practices and the adoption of new solutions, while also contributing to making solutions more sustainable [63]. Secondly, some participants have been involved in the participatory process over the long term and some of them were even already engaged in changing their practices [36]. Local authorities were involved from the beginning through meetings with the research team to understand the issues regarding AMR faced in the province and to obtain the authorizations. They were engaged in developing our survey, identifying participants, and disseminating results, which led to a greater engagement. One of the organizational design principles is to recognize that some stakeholders can play a key role in the process, involving the stakeholders in research design and recognizing the key role that they can play in it, which strengthens stakeholders’ engagement [62]. Indeed, gathering different types of stakeholders can lead to the identification of leaders in changing practices. For example, the leader of the cooperative and the veterinarian working at the district veterinary station were two key stakeholders in this area. During the workshops, they showed leadership capabilities and could be considered as “champions” in the change of practices. Finally, we organized a restitution meeting a year later to validate our results and maintain the commitment of stakeholders to changing their practices.

This method helps to identify pathways to reach a common interest (i.e., ABU reduction) and develop adapted solutions [64] by bringing together people working in multiple sectors (public, private, academic) and at different levels. In our case, some interactions already existed before the workshops. However, bringing these actors together could strengthen their relations and create a new network of stakeholders. This approach stimulates the discussion between different stakeholders with various opinions, practices, and knowledge regarding antibiotic usage. Confronting opinions with one another allows the emergence of new forms of knowledge, ideas, and solutions. Thus, it has been interesting to stress the need for training closer to reality. Participatory methods also increase the acceptability of measures when they are designed by the concerned stakeholders [65].

Based on the three workshops, we have built the impact pathway to explain our theory of change. The long-term impacts identified were a reduction in AMU and improvements in farming practices. This mapping illustrates the solution process by stressing the causal relationship between inputs and the desired impacts of the solutions developed [46]. But we can also think about the impact of the transdisciplinary co-construction process. Schneider et al., identified the outcomes of such transdisciplinary process as the co-production of new knowledge, the development of shared understanding and new competencies, including potential impacts of more informed and equitable decision-making, collective action, and reflective leadership, leading to sustainable transformations [66]. The social learning of these approaches induces that shared knowledge leads to collective action [27].

### Practical challenges of co-construction approaches

The selection of the participants and the quality of the facilitation are some of the main keys to the success of such processes [27,61]. In our case, half of the participants already knew the research team and the pre-existing relation of trust facilitated their participation. However, some other participants, particularly farmers or local drug sellers did not express their opinion as often during the discussions. We tried to include them, by asking specific questions to these stakeholders. This is a good way to understand their opinion but can make the participants uncomfortable if they do not want to speak. It could have been interesting to conduct face-to-face interviews as has been done in a similar study in France [28]. Power dynamics between participants can make it challenging to manage discussions and integrate everyone’s ideas. Indeed, it has been demonstrated that social learning in the planning process (i.e., interactions between actors) that leads to knowledge exchange and knowledge development can have positive or negative impacts that need to be kept in mind [67]. That is why a good balance needs to be found between officials and private stakeholders as well as between stakeholders operating at provincial or communal levels to help everyone participate. Related to this, social desirability bias might have occurred during our study since the participants were aware of the study’s objectives. Participants could have agreed to statements to match what was expected from them (i.e., the need to reduce the usage of antibiotics, the production of chickens with less antibiotic use, better availability of alternative products, or the need to be trained to sell and use antibiotics) rather than expressing their true feelings and opinions [68]. In our study, household farms were targeted by the participants as responsible for antibiotic misuse because of a lack of biosecurity, high density of farms, and poor knowledge and willingness to change. Other surveys have already shown that household farmers misused antibiotics in a similar way to family commercial or intensive farmers [42,69]. However, they still represent the most common production system in Vietnam, linked to consumers’ habits of buying live birds in local markets and are an important part of the livelihood of the local population. Because no household farmers were invited to participate in the workshops, we could not gather their opinion on this point. Our results could have been slightly different because some participants could have been reluctant to identify them as the main culprits in the misuse of antibiotics. The facilitation needs also to be considered carefully. Facilitators must be trained before the workshops on participatory methods. In our case, one of the facilitators couldn’t attend the second workshop and had to be replaced. This should be limited while doing such methods as each workshop is connected to another.

While our methodological framework is based on a well-described process, it remains inherently inductive and flexible [46]. The format, which usually takes place in three full days had to be adapted to the constraints of the participants. Some steps were also shortened and simplified. Indeed, we were only able to explore one of the barriers and develop solutions for this issue. Some steps were also more challenging than others. Building the common vision and identifying obstacles to its realization were steps easily understood. However, outcome mapping was a difficult step for the participants. They had to identify changes that must be made to overcome the problems, but then they had to identify new obstacles that prevent these changes from happening. For our study, we should have simplified this step, which would have allowed us more time to discuss the solutions. It is also difficult to assess whether the participants have understood the process or not. Indeed, some of them expected us to provide concrete solutions. We tried to limit this by clearly explaining the objectives of the study. Finally, we found it difficult to re-validate the data collected during a previous phase by the participants, as they considered it already finalized.

A process like this is not always evaluated, and the designed strategies are not always implemented [27]. In our case, questionnaires were given to the participants before the first workshop and after the last workshop. The first questionnaire aimed at understanding participants’ expectations, their understanding of ABU and ABR, and their involvement in changing practice. The second one aimed to assess the satisfaction of the participants regarding their participation to the workshops, knowledge improvement on ABU and ABR, and the following steps they would like to take regarding ABU reduction. One of the main outputs of this short evaluation survey was their satisfaction to have participated in the workshops. They also recognized that they had gained knowledge during the three workshops, even if it was not clear if this was more related to ABU or its reduction. To reach a credible evaluation of the process, we should have designed it more carefully and included it as part of our study objectives [70]. The evaluation of the co-design strategies can be performed by translating the results (shared vision and impact pathway) into an outcome-oriented monitoring and evaluation system prior to the implementation of the strategies. This system, which can be participative, focus on monitoring the outcomes to document the change processes rather than focusing only on the outputs [71,72]. One year later, no concrete actions have been taken in the commune, which can be partly explained by the fact that many of the recommendations do not depend on the communal level. This also shows the challenge of maintaining stakeholders’ engagement after the end of a research project if no one is taking the lead. Although one of our objectives was to develop an Action Lab in this commune, time and budget constraints prevented its realization. However, the key stakeholders have been introduced to other research institutes that aim at developing another research project in the region.

### Impact on policy and future directions

In our survey, farmers, drug sellers, and local veterinarians were able to interact with researchers and stakeholders operating at district and provincial levels for public services. In Vietnam, the provincial veterinary services represent the link between the communal and the national level [48]. They can help to scale up results and implement innovations locally. Because of their central position in the flow of information and the chain of command, according to Binot et al., they should now be integrated into a multi-level integration process to scale up the results and develop national policies by using participatory modelling [73].

The study’s findings have important implications for national policy, particularly given Vietnam’s ongoing efforts to combat AMR [44]. By identifying practical, community-driven strategies to reduce antibiotic use in chicken production, this study contributes to the objective of raising awareness about AMR but also by promoting good ABU practices in livestock production system outlined in the Vietnamese National Action Plan on AMR [44] and provides a model that could be replicated across the country in other provinces. One of the study’s key recommendations is the implementation of targeted training programs in biosecurity and organic farming practices. These could be integrated into national agricultural extension services, with specific policies designed to provide financial and technical assistance to farmers and drug sellers who participate in these programs. Furthermore, the success of the participatory approach used in this study suggests that future AMR strategies should place a greater emphasis on stakeholders’ engagement and community involvement, potentially leading to more sustainable and widely accepted practices.

One of the limitations of the participatory process resides in its local nature that leads to the design of solutions that without high level of support (political will and financing), will be unlikely to be implemented. Moreover, most of our participants were not AMR experts. The solutions designed were thus not done considering the scientific literature on transforming behaviors in relation to AMU. The co-design process would have benefited from clearer scope and feasibility factors. However, our approach complements both top-down and bottom-up actions, as local ideas must be linked to meso-level actors (such as district/provincial veterinary services, producer associations, retailers), who were included in our study and who can then mobilize resources and harmonize policies. To ensure that these findings are translated into effective policy, it is critical to engage policymakers throughout the implementation process. Collaboration among research institutions, government agencies, and local communities will be critical for developing policies that are both practical and effective.

## Conclusion

Participatory approaches through impact pathway assessment are valuable tools for identifying strategies to address complex health challenges such as AMR. By engaging diverse stakeholders, including farmers, drug sellers, policymakers, and research institutions, this study successfully designed community-driven solutions tailored to local contexts for reducing antibiotic use in chicken production in Vietnam. This collaborative process has highlighted critical barriers, such as limited awareness and training on biosecurity and organic farming and proposed actionable strategies to overcome them. By involving local participants in every stage, from defining the vision to mapping outcomes and formulating action plans, the approach ensured that solutions were context-specific and widely accepted. For example, participants identified key strategies like creating training programs closely aligned with field realities and launching communication campaigns via social media and television to reach a broader audience.

The proposed action plans, including practical training for animal health professionals and communication initiatives, represent a model for reducing antibiotic misuse that can reach a large audience. However, the study also revealed systemic challenges, such as economic constraints, entrenched farming practices, and limited enforcement of organic farming standards, which require continued focus and policy support. Despite promising outcomes, maintaining stakeholder engagement post-research and implementing immediate strategies remain challenging. To bridge this gap, it is essential to integrate the findings into national policies, such as Vietnam’s National Action Plan on AMR. Policymakers, in collaboration with academic and local institutions, should prioritize financial and technical support for scaling these initiatives while ensuring alignment with broader public health goals.

In conclusion, this research demonstrates the transformative potential of participatory methods in tackling AMR challenges in livestock production. By fostering local ownership and building trust among stakeholders, these approaches pave the way for more effective, inclusive, and sustainable solutions. Future efforts should focus on scaling these interventions, enhancing cross-sector collaboration, and continuously refining strategies based on monitoring and evaluation to ensure long-term impact on AMR mitigation. Community-driven solutions should then be scale-up at the national level and integrated into national action plan. This will also contribute placing a greater emphasis on stakeholders’ engagement in AMR policies.
